# Reward-representing D1-type neurons in the medial shell of the accumbens nucleus regulate palatable food intake

**DOI:** 10.1038/s41366-018-0133-y

**Published:** 2018-06-15

**Authors:** Máté Durst, Katalin Könczöl, Tamás Balázsa, Mark D. Eyre, Zsuzsanna E. Tóth

**Affiliations:** 1 Laboratory of Neuroendocrinology and In Situ Hybridization, Department of Anatomy, Histology and Embryology, Semmelweis University, Tűzoltó utca 58, Budapest, Hungary; 2 Department of Physiology I, University of Freiburg, Hermann-Herder-Str. 7, Freiburg, 79104 Germany

**Keywords:** Obesity, Obesity

## Abstract

**Background/objectives:**

Dysfunction in reward-related aspects of feeding, and consequent overeating in humans, is a major contributor to obesity. Intrauterine undernutrition and overnutrition are among the predisposing factors, but the exact mechanism of how overeating develops is still unclear. Consummatory behavior is regulated by the medial shell (mSh) of the accumbens nucleus (Nac) through direct connections with the rostral part of the lateral hypothalamic area (LHA). Our aim was to investigate whether an altered Nac-LHA circuit may underlie hyperphagic behavior.

**Subjects/methods:**

Intrauterine protein-restricted (PR) male Wistar rats were used as models for hyperphagia. The experiments were performed using young adult control (normally nourished) and PR animals. Sweet condensed milk (SCM) served as a reward to test consumption and subsequent activation (Fos+) of Nac and LHA neurons. Expression levels of type 1 and 2 dopamine receptors (D1R, D2R) in the Nac, as well as tyrosine hydroxylase (TH) levels in the ventral tegmental area, were determined. The D1R agonist SKF82958 was injected into the mSh-Nac of control rats to test the effect of D1R signaling on SCM intake and neuronal cell activation in the LHA.

**Results:**

A group of food reward-representing D1R+ neurons was identified in the mSh-Nac. Activation (Fos+) of these neurons was highly proportional to the consumed palatable food. D1R agonist treatment attenuated SCM intake and diminished the number of SCM-activated cells in the LHA. Hyperphagic PR rats showed increased intake of SCM, reduced D1R expression, and an impaired response to SCM-evoked neuronal activation in the mSh-Nac, accompanied by an elevated number of Fos+ neurons in the LHA compared to controls.

**Conclusions:**

Sensitivity of food reward-representing neurons in the mSh-Nac determines the level of satisfaction that governs cessation of consumption, probably through connections with the LHA. D1R signaling is a key element in this function, and is impaired in obesity-prone rats.

## Introduction

Feeding reward dysregulation is a basic factor in overeating and consequent obesity [1]. The central process leading to reward dysregulation is still unknown, but both low and high birth weights are implicated as risk factors [2, 3]. Rats prenatally exposed to scarcity regarding overall nutrition, or merely protein restriction, show profound hyperphagia [4, 5] and an altered behavioral response to feeding reward [6, 7]. Reward deficit — reduced reward from eating and continued food intake to reach satisfaction — has been suggested as an underlying factor in both humans and rodents, which was connected to striatal type 2 dopamine receptor (D2R) downregulation and hypofunction [8–10]. However, according to other data, D2R downregulation cannot be a primary factor in hyperphagia, but instead is a consequence of overconsumption, increased body mass index and leptin levels [8, 11, 12].

Palatable food intake is regulated mainly by the accumbens nucleus (Nac) circuits [13]. Most of the neurons in the Nac (95%) are medium spiny neurons (MSNs) primarily bearing D1Rs and/or D2Rs, and receiving dopaminergic input from the ventral tegmental area (VTA) [14]. Subregions of the Nac are distinguished as the shell and the core [15], which participate in different aspects of feeding reward in rats. Using Fos protein induction it was shown that the actual pleasure (palatability or ‘liking’) of food consumption is represented within the cells of the rostral dorsomedial portion of the shell (i.e. medial shell (mSh)) [16], while the motivation to eat, or incentive salience (‘wanting’), is represented within the mSh, as well as in the entire core [17]. The mSh has been implicated in the intimate control of feeding behavior, as blockade of glutamate receptors exclusively here, but not in the core, elicits pronounced feeding [18]. Stopping feeding, which is crucial to prevent overeating, is also realized via a pathway originating from the mSh [19, 20]. The pathway includes D1R-expressing MSNs that, upon activation, stop ongoing eating in mice [19]. These D1R+ neurons target the rostral part of the lateral hypothalamus (LH) [20, 21], an area known to be engaged in reward-related food intake regulation [13]. The lateral hypothalamic area (LHA) target neurons increase their calcium activity during the eating of palatable food, and are distinct from the mostly more caudally located melanin-concentrating hormone (MCH) and orexin producing neurons [21, 22].

We hypothesized that a low protein diet during fetal development leads to long-term changes in Nac-circuits and affects mechanisms involved in the cessation of eating. To test this, we compared the hedonic food intake of satiated rats with or without prenatal protein restriction. We evaluated the microstructure of reward-related consummatory behavior and analyzed the Fos expression elicited by palatable food in the Nac and the LHA. We found that the activation pattern of D1R+ MSNs in the mSh reflects the reward value of food, and that altered D1R signaling probably plays a decisive role in hyperphagic behavior.

## Materials and methods

### Animals

Rats (Wistar, Toxi-Coop Toxicological Research Center Zrt, Dunakeszi, Hungary) were housed under standard laboratory conditions (22 ± 1 °C, 12-hour day cycle) and had free access to standard rodent chow and tap water except when otherwise indicated. When anesthesia was needed, a mixture of ketamine (75 mg/bwkg) (Richter Gedeon Nyrt, Budapest, Hungary) and xylasine (15 mg/bwkg) (CP-Pharma, Burgdorf, Germany) was injected intramuscularly. Experiments were performed according to the European Communities Council Directive (86/609/EEC/2 and 2010/63/EU) and were supervised by the Animal Care and Use Committee of the Institute of Experimental Medicine, Hungarian Academy of Sciences, Budapest, Hungary (XIV-I-001/2262-4/2012).

Experimental design and sample sizes are shown in Supplemental Table (ST) 1. Experiments were performed once, except otherwise indicated.

### Intrauterine feeding protocol

Timed pregnant female rats were housed individually and kept on a low protein diet (8.8% crude protein, 8.1% crude fat, 5.0% crude fiber, 5.3% crude ash, 69.7% nitrogen-free extracts (Ssniff Spezialdiäten GmbH, Soest, Germany, catalog# E15202-24)) only during pregnancy. Control timed pregnant female rats received standard laboratory chow (19.2% crude protein, 4.1% crude fat, 6.1% crude fiber, 6.9% crude ash, 53.4% nitrogen-free extracts (Altromin Spezialfutter GmbH, Lage, Germany, catalog# 1324)). Energy in the low protein diet was made up with additional carbohydrate and lipid. More details on diets are provided in ST2, and the full compositions can be downloaded from the relevant Company home page (http://www.ssniff.com and https://altromin.com). After delivery, protein-restricted (PR) dams were returned to the standard laboratory chow diet. To standardize nursing conditions all litter sizes were adjusted to eight male pups (no females). Control and intrauterine PR dams nursed control and intrauterine PR pups, respectively. Bodyweight and food intake after weaning were measured weekly for each animal. Experiments were carried out using these male offspring at 14 weeks of age. Experimental groups (control and PR) consisted of subjects of three different litters to avoid any intra-litter effects or bias.

### Assessment of palatable food intake

The influence of palatable food was examined by presenting sweetened condensed milk (SCM; Sole-Mizo Zrt, Csorna, Hungary) (diluted 1:2 in tap water) to control and intrauterine PR animals, according to the protocol of Brand et al. [23]. Briefly, this protocol was in three stages. Firstly, the evening prior to the training day, rats in their home cage were presented with a water bottle containing SCM and allowed a few licks in order to familiarize the animals to the taste of SCM. Next morning, at the beginning of the passive phase, when animals were sated after nocturnal food intake, rats were transferred to the experimental room suitable for behavioral recordings. They were placed into a clean “experimental” cage, to familiarize them with the behavioral recording environment for 5 min. SCM was then presented for a further 10 min, and consumption was measured. Finally, the next (experimental) day, rats were assigned into experimental sessions. For every session, one pair of rats was investigated, selected randomly from different experimental groups. The experimental protocol was the same as the previous training day, but the behavior was also video recorded, and used for analysis. Animals were placed back to their home cages for 90 min, and then anaesthetized and transcardially perfused with 4% paraformaldehyde in 0.1 M phosphate buffered saline. Brains were removed, post-fixed for 1 h in the same fixative, immersed in 20% sucrose solution overnight, and then frozen and stored at −80 °C until processed.

### D1R-agonist treatment

Normally nourished animals were handled daily for 3 weeks before experiments. One week before the experiments they were implanted with a chronically indwelling guide cannula (Plastics One Inc, Roanoke, VA) (22 ga) directed 0.5 mm above the right Nac mSh (1.48 mm rostral to bregma, 0.82 mm lateral to midline, 6.47 mm ventral from the skull) [24]. Rats were kept individually after operation. Reward-driven behavior was assessed after recovery, using the same training schedule as described above, except that 10 min before SCM was provided, rats received intra-accumbal drug injections. The different treatment groups received 0.5 or 3 µg of the selective D1 receptor agonist SKF-82958 (Sigma-Aldrich Kft.; Budapest, HU) dissolved in 1 µl of physiological saline, or vehicle alone, through internal cannulas with 0.5 mm extensions beyond the guide cannulas. Cannula placements were evaluated after perfusion fixation on histological brain sections. Only animals with correct cannula positions and uninjured lateral ventricles were included for further analysis.

### Microstructure analysis of feeding behavior

Behavioral video recordings were slowed down to 0.25 times normal speed (VLC media player, VideoLAN, Paris, France) during analysis to achieve sufficient resolution in time. Meal duration, mean size and duration of lick clusters, and initial & mean rates of licking were determined for each group. The effective time spent licking during the 10 min experimental period was regarded as meal duration. By definition, continuous segments of licking separated by pauses longer than 500 ms were regarded as lick clusters [25]. The number of lick clusters was determined and used to calculate mean cluster duration (meal duration/cluster number) and cluster size (cluster duration × mean licking rate). Mean licking rates were determined by counting the number of licks during six periods (6 × 10 sec) of continuous licking that were selected equally across the whole drinking process. Initial licking rates were calculated based on data from the first period chosen at the beginning of the drinking process.

### Immunohistochemistry

Single Fos, Fos–orexin, and Fos–MCH double immunostainings, as well as D1R immunofluorescence staining, were applied on sections from different experiments (see ST1). Standard immunohistochemical reactions were performed using 50 µm thick, perfused-fixed, free-floating sections as described previously [26], except for the following reactions: (1) for Fos immunostaining prior to in situ hybridization, a modified serum-free protocol was applied using 20 µm-thick sections. Solutions were treated with 0.1% diethylpirocarbonate, and 1000 U/ml heparin was added to inhibit RNases (Sigma), (2) for D1R protein visualization we used 50% ethanol application for 30 min instead of our standard permeabilization with 1% Triton X-100 (Sigma). For more details on antibodies and detection, see ST3.

### In situ hybridization

Sections mounted on Superfrost Ultra Plus slides (Thermo Fisher Scientific, Waltham, MA, USA) were hybridized using S35-UTP-labeled riboprobes to detect D1-2R mRNAs in the Nac, and tyrosine hydroxylase (TH) precursor mRNA in the VTA, as described previously [27]. Riboprobes were prepared using D1R (GenBank Acc: NM_012546.3, 981–1393 bps) and D2R (NM_012547.1, 981–1393 bps) cDNA fragments subcloned into Bluescript KSII + vectors as templates. Specificity of cDNAs was verified by sequencing and assessed by BLAST screening (https://blast.ncbi.nlm.nih.gov/Blast.cgi) of the rat genome. The rat tyrosine hydroxylase (TH) intronic cDNA was provided by Harold Gainer (NIH, Bethesda, MD) [28]. After hybridizations, sections were apposed to a BAS-MS imaging plate (Fuji Photo Film Co., Ltd., Kanagawa, Japan, NJ) for 2 (DRs) and 7 (TH) days, and then data were read out by a Fujifilm FLA-8000 Image Analyzer. Sections with D1R and D2R labeling were dipped in Kodak NTB nuclear emulsion (Carestream Health Inc., Rochester, NY) for 5 days according to the manufacturer’s instructions and developed using Kodak developer and fixer (Sigma) [29, 30].The sections were stained with Giemsa (Sigma) except those with prior Fos immunohistochemistry.

### Quantitative analyses of immunostained sections

Evaluations were performed using microphotographs taken by an Olympus BX60 microscope (objective: UPlan FL 4×/0.13) interfaced with a SPOT Xplorer 17.4 camera (Diagnostic Instruments Inc., Sterling Heights, MI) and with the help of the ImageJ 1.46r (Wayne Rasband NIH, Bethesda, MD) application.

Fos-labeled cells in Nac and in the LHA were counted within regions of interest bilaterally (ROI sizes: 200 µm × 200 µm and 400 µm × 400 µm for Nac and LHA, respectively). The Nac was evaluated in three sections/animal representing the rostral part of the Nac between [1.7–2.2 mm] rostral to Bregma [24]. The core was evaluated as the sum of two regions (ROI 1 and 2), ROI 3 was superimposed over the mSh (hedonic hot spot [16]), ROI 4 was placed ventrally to the mSh. The LHA was evaluated in five sections/animal representing the area [1.8–2.4 mm] caudal from Bregma [20]. ROI 5 was superimposed lateral to the fornix (referred as “perifornical” region, PF), ROI 6 was overlaid dorsal to the optic tract representing the lateral hypothalamus (LH) (see illustrations in the Results section). The number of cells/animal was used for statistics.

The optical densities of D1R immunostained sections were measured according to Sato et al. [31] in the mSh and core on gray scale unmodified images in three sections/animal using the same ROIs as above. The average/animal data were used for statistics.

### Quantification of in situ hybridization data

Radioactive in situ hybridization provides a linear relationship between the signal intensity and the mRNA expression level [32]. D1-2R mRNA expressions were determined from images of autoradiographic emulsion-coated sections, as earlier described [33, 34]. Microphotographs were taken by an Olympus BX51 microscope (objective: UPlan FLN 10×/0.30) attached to a QImaging QCam system (Quantitative Imaging Corporation, Surrey, Canada). Two images were taken/area. The darkfield image was superimposed on the brightfield image. The brightfield images were used to identify cells. Measurements were performed on the darkfield images. The labeled cellular profiles were selected using the threshold tool in the ImageJ software. The area covered by silver grains was measured and expressed as pixels/cell [34, 35]. Expression level of TH precursor mRNA in the VTA was evaluated from the image recorded by the phosphoimager as earlier described [36]. Mean gray values were measured bilaterally with ImageJ, in three sections/animal (D1R) between [1.7–2.2 mm] rostral to Bregma, and in six sections/animal (TH) between [5.0–5.4 mm] caudal from Bregma. In all cases, the average/animal data were compared statistically.

Fos immunoreactivity and D1R-mRNA labeling in the Nac mSh were counted similarly as described above using microphotographs (taken using an Olympus BX60 microscope (objective: UPlan 20×/0.50 Ph1). Percentages of single-labeled and double-labeled cells were calculated.

### Statistics

Data analyses were performed by investigators blinded to treatments. Statistical significances were calculated employing Sigmastat 3.5 application (Systat Software, Inc., Chicago, IL). Studies were designed to reach the desired power of 0.8 with alpha = 0.05. For comparing control and PR groups Student’s *t*-tests (two-tailed) were used. When treatment (intrauterine conditions) and time interaction was also evaluated, repeated measures ANOVA was used. For evaluating effects of different doses of D1 agonist treatment, a one-way ANOVA test followed by Student–Newman–Keuls post-hoc analysis was applied. Correlations were calculated by the Pearson method. When normality or equal variance tests failed, non-parametric equivalents of the above methods (Mann–Whitney test, Kruskal–Wallis test, Spearman method, respectively) were used. Differences between groups were considered statistically significant when *p < 0.05. Results are expressed as means ± SEM values.

## Results

### Development of intrauterine PR animals

**Fig. 1:**
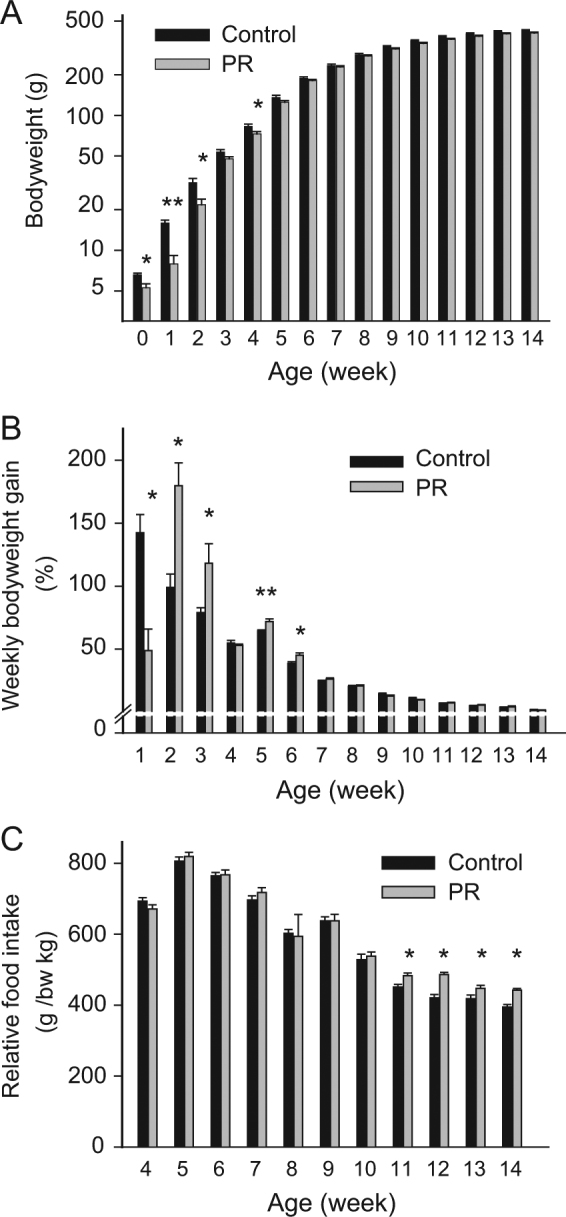
Bodyweight and food intake during postnatal development. **a** Weekly bodyweight measurements. PR pups were born smaller. The difference between groups diminished by the 6th week. **b** Weekly bodyweight gain relative to the bodyweight measured on the previous week. There was a decrease with age in both groups (*p* < 0.001). PR pups gained poorly during the 1st week. They gained relatively more weight than controls in the next few weeks (catch up growth). **c** Food consumption relative to bodyweight, measured weekly after weaning. PR rats consumed relatively more of chow than controls starting from the 11th week. Data are presented as means ± SEM. *n* = 9-8, **p < 0.01, *p < 0.05 vs. controls

The low protein diet did not influence the duration of pregnancy (control: 22.6 ± 0.2 days vs. PR: 22.3 ± 0.3 days) or the litter size (control: 15.1 ± 0.9 vs. PR: 14.6 ± 0.6 pups). It resulted, however, in a significant reduction in the birthweights of offspring (Fig. 1a, *p* < 0.05). The bodyweight of PR pups was markedly lower compared to controls, especially on the first (*p*< 0.01), but also on the second postnatal week (*p* < 0.05). Subsequently, the PR animals started to catch up controls and the bodyweights were comparable from the 5th week onward. From the 11th week to the time of the experiments (14th week), bodyweights of PR and control peers did not differ significantly (Fig. 1a). The weekly body weight gain decreased with age in both groups (*p* < 0.01) (Fig. 1b). During the 1st postnatal week PR pups fell behind controls in weight gain (*p* < 0.05). They then exhibited a period of accelerated weight gain from the 2nd to the 6th week to catch up controls (2nd, 3rd, 6th weeks *p* < 0.05, 5th week *p* < 0.01). The relative bodyweight gain of groups was similar later on. Food intake of rats did not differ in the first 10 weeks, but from the 11th week PR animals became hyperphagic (Fig. 1c, *p* < 0.05)

### Palatable food consumption

In order to study feeding reward, a highly palatable food, sweetened condensed milk solution, was offered to satiated animals. PR subjects consumed more SCM during the training day (6.16 ± 0.9 and 15.11 ± 1.3 g for control and PR groups, respectively; *p* < 0.001), and also on the experimental day (*p* < 0.05) (Table 1). Microstructure analysis of eating behavior showed a slight, non-significant increase in meal duration (*p* = 0.071), and no difference in initial and mean licking rates, mean duration or size of lick clusters compared to controls (Table 1). As lick cluster size and initial licking rate measure hedonic response to palatability (‘liking’) [25, 37], we concluded that PR rats did not exhibit a heightened hedonic response.

**Table 1:** Palatable food intake and microstructure of consummatory behavior of control and intrauterine protein-restricted (PR) rats

	SCM intake (g)	Meal duration (s)	Initial licking rate (1/s)	Mean licking rate (1/s)	Mean cluster size (licks)	Mean cluster duration (s)
Control	9.5 ± 1.1	342 ± 40	6.3 ± 0.1	6.2 ± 0.1	59.3 ± 5.6	9.6 ± 1.0
PR	14.1 ± 1.2*	441 ± 28	6.2 ± 0.0	6.0 ± 0.1	61.6 ± 7.3	10.2 ± 1.2

Data are presented as means ± SEM, *n* = 8–9 for sweet condensed milk (SCM) intake and *n* = 7–8 for all other parameters

*p   < 0.05

### Impact of feeding reward on Fos induction in the Nac and rostral LHA

**Fig. 2:**
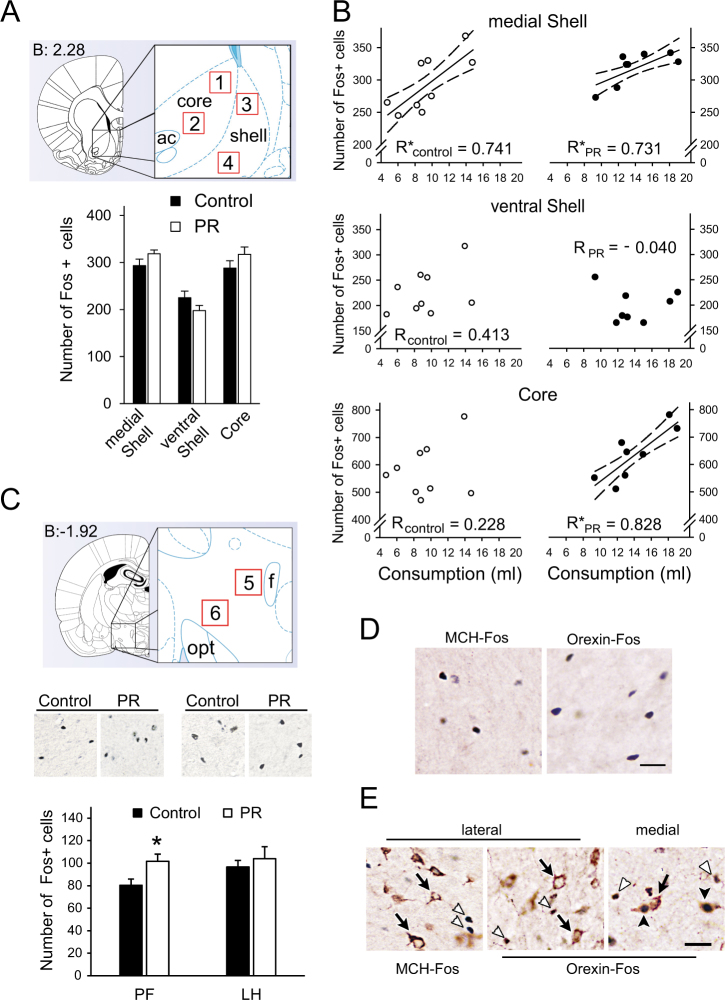
Fos activation pattern of accumbal and lateral hypothalamic neurons following intake of highly rewarding food. **a** Top: Brain regions of interest (ROIs) for counting Fos-labeled cell nuclei in the accumbens nucleus. The diagram was adopted from the Atlas of Paxinos and Watson [24] and illustrates a sampling frontal section level of 2.28 mm to Bregma. ROIs (200 µm × 200 µm) were overlaid on microphotographs in the different areas: 1–2, core; 3, medial shell hedonic hot spot; 4, control area in the ventral part of the shell. Bottom: The number of Fos-positive cells per area/animal. No significant difference existed between the experimental groups, *n* = 8-9. **b** Fos activation in the accumbens nucleus in relation to the amount of consumed sweet milk. Strong correlations were found in the medial shell (top) in both control (left, open circle) and PR (right, filled circle) groups and in the core (bottom) in PR rats only. Note that Fos-counts are in the same range in the individual areas, but there is a shift toward higher quantities of consumed milk in PR rats. Solid and dashed lines show the regression lines and the 95% confidence intervals, respectively, see correlation coefficients (*R*) on the graphs, *p < 0.05, *n* = 8–9. **c** Top: a similar diagram as in A, showing location of ROIs (400 µm × 400 µm) for counting Fos-labeled cell nuclei in the rostral lateral hypothalamic area at a sampling frontal section level of −1.92 mm caudal from Bregma. ROIs were placed: 5, laterally to fornix (PF), 6, on the lateral hypothalamus (LH). Bottom: The number of Fos+ cells per area/animal. A higher Fos-count was detected in the PF area in PR rats, but there was no significant difference in the LH region. Fos+ cell nuclei appear as black dots on the representative pictures (left the PF, right the LH regions, respectively, *n* = 7,*p < 0.05 vs. controls. : **d** Palatable food intake-activated neurons in the rostral LHA were non-MCH, non-orexin cells. Double immunostainings from control animals. Fos+ nuclei are seen as dark dots. Scale: 50 µm. **e** Fos induction in the caudal part of the hypothalamus. Many Fos+ cells were seen caudal to the analyzed area (white arrowheads). MCH cells were Fos-negative. Orexin cells lateral to the fornix were also Fos-negative. Many of orexin cells were double-labeled medial to the fornix. Black arrows: Fos-negative MCH or orexin cells, black arrowheads: orexin and Fos double-labeled cells. Control animals. Scale: 50 µm. Data are presented as means ± SEM. ac anterior commissure, f fornix, LH lateral hypothalamic, and PF perifornical regions of the lateral hypothalamic area (LHA), opt optic tract, PR intrauterine protein-restricted

Neuronal activation elicited by SCM consumption in the Nac was measured by counting the number of Fos+ neurons in the core (associated with ‘motivation’) (Fig. 2a, ROIs 1. and 2.), in the mSh hot spot (associated both with ‘motivation’ and ‘liking’) (Fig. 2a, ROI 3.) and ventral to this area within the shell (no currently known association) (Fig. 2a, ROI 4.) identified according to coordinates given by Pecina and Berridge [16]. The average Fos**-**count was similar between groups in all of the investigated areas (Fig. 2a, bottom). To see how reward-related consumption is reflected in cell activation in Nac, relationships between the volumes of consumed SCM and the Fos**-**counts were examined *within* groups. Both controls and PRs produced strong, linear, positive correlations within the mSh (*R*_Control_ = 0.741, *p* < 0.05; *R*_PR_ = 0.731, *p* < 0.05). However, we noticed a right shift toward higher amounts of SCM consumption in PR rats compared to controls (Fig. 2b top). No correlations were found in the control area, in the ventral shell (*R*_Control_ = 0.413, *p* = 0.269; *R*_PR_ = −0.040, *p* = 0.925) (Fig. 2b middle), and Fos**-**count and SCM intake correlated positively in the core in PR rats only (*R*_Control_ = 0.228, *p* = 0.556; *R*_PR_ = 0.828, *p* < 0.05) (Fig. 2b bottom). Thus, (1) reward valence of food is strictly encoded as a “Fos-print” within the mSh; (2) mSh neurons in PR rats have a heightened activation threshold for palatable food intake; (3) there is a difference between PR and control rats in the reaction for reward-related food consumption in the core.

Neuronal activation in response to SCM intake was also determined in the target area of mSh neurons in the *rostral* part of the LHA. Based on earlier findings, ROI analysis was performed in two regions [20]; lateral to the fornix (perifornically, PF) (Fig. 2c top, ROI 5.) and in the lateral hypothalamus (LH) (Fig. 2c top, ROI 6.). PR animals with higher SCM intake exhibited more Fos+ cells than the controls in the PF region (ROI 5, *p* < 0.05) (Fig. 2c, bottom left). The LH area (ROI 6) responded to the challenge similarly in both groups (Fig. 2c, bottom right). MCH-producing and orexin-producing cells were not present at this rostro-caudal level (Fig. 2d). MCH and orexin neurons caudal to the analyzed area were not Fos+ lateral to the fornix, while many orexin cells were Fos+ medial to the fornix (Fig. 2e).

### Evaluation of the main components of the dopaminergic transmission to the Nac

**Fig. 3:**
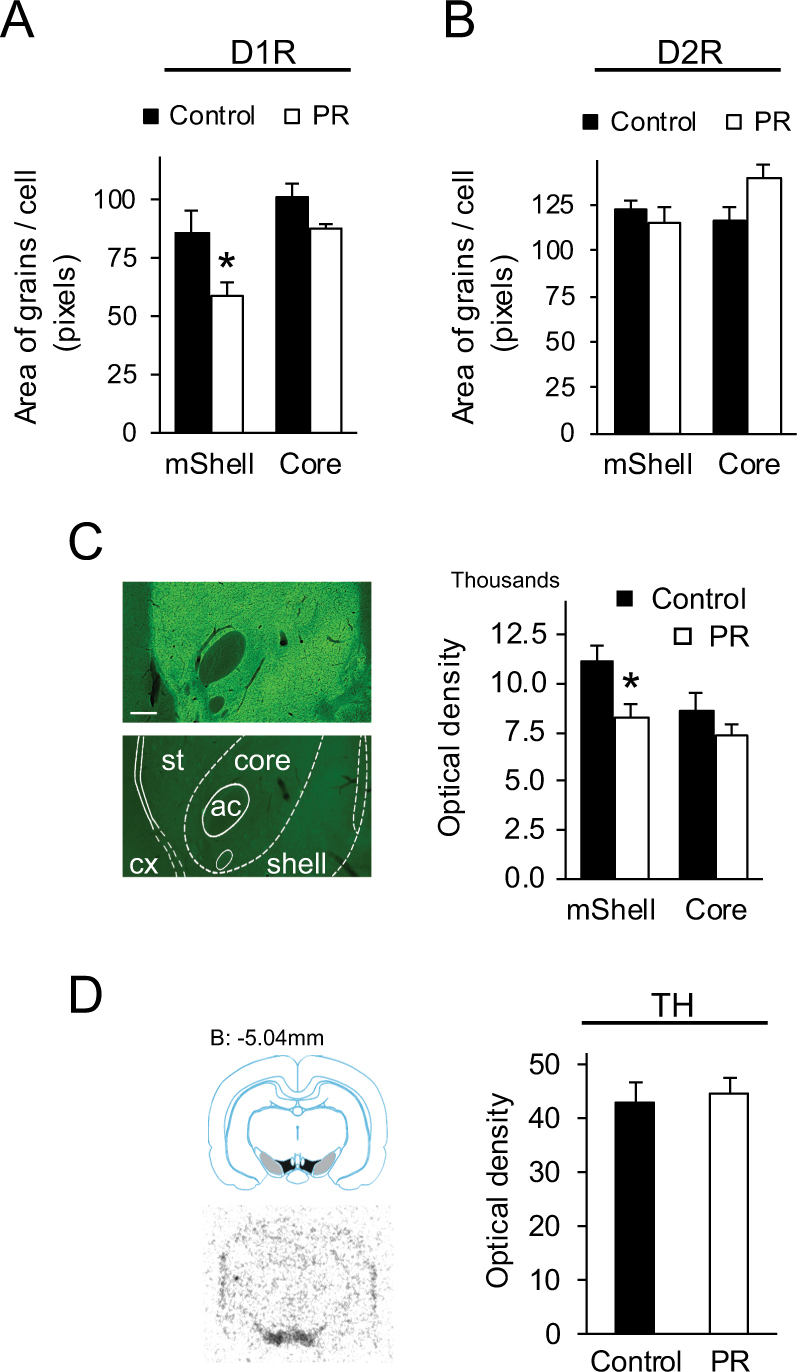
Expression of key molecules of dopaminergic transmission in non-challenged rats. **a** D1R mRNA expression in the accumbens nucleus decreased significantly in the medial shell in PR rats. **b** D2R mRNA expression did not change significantly. **c** D1R protein expression. There was an intense labeling by immunohistochemistry in the striatum and the accumbens nucleus compared to the cortex (left, top). No signal was detected in sections when the primary antibody was omitted (left, bottom). Scale: 250 µm. Right: Intensity measurements of immunostainings confirmed the reduced D1R protein expression in the medial shell in PR rats. There was no significant difference in the core. **d** Expression of tyrosine hydroxylase precursor mRNA in ventral tegmental area (VTA). Left, top: A diagram adopted from the Atlas of Paxinos and Watson [24] illustrating a sampling frontal section level of 5.04 mm caudal from Bregma. At this level TH is expressed in the VTA (black) and the substantia nigra (gray). Left bottom: Autoradiographic image of a section from the corresponding level, showing intense precursor TH mRNA expession according to the labeled areas above. Only a background is seen in the other parts of the section. Optical density measurements in the VTA showed that TH gene expression was unaffected by prenatal nutritional conditions. Data are expressed as means ± SEM, *n* = 6, *p < 0.05 vs. controls. ac anterior commissure, cx cortex, mShell medial shell, PR intrauterine protein-restricted, st striatum, TH tyrosine hydroxylase

Substantially less D1R mRNA was expressed in the neuronal somata in the mSh in PR animals (*p* < 0.05), but expression in the core was similar to controls (Fig. 3a). Unlike the D1R, expression of D2R mRNA was not significantly different between groups (Fig. 3b). Immunohistochemistry showed intense expression of D1Rs in the striatum and the Nac compared to the cortex (Fig. 3c left, top). D1R immunostaining appeared as seemingly homogenous labeling; the cells, dendrites, and axons were indistinguishable, in accordance with earlier data [38]. There was no specific staining in the sections when the primary antibody was omitted from the reactions (Fig. 3c left, bottom). Optical density analysis confirmed the reduced D1R protein expression in the mSh (*p* < 0.05), a difference that was not apparent in the core (Fig. 3c, right).

To assess whether D1R density is related to altered dopamine production, we measured TH expression in the VTA, as this provides the main dopaminergic input to the Nac. Since there is a large pool of both TH mRNA and protein here, we detected TH precursor mRNA expression (Fig. 3d left), which reflects the rate of transcription directly. Based on our data, TH gene expression was unaffected by prenatal feeding conditions (Fig. 3d, right).

### Role of mSh D1Rs in reward-driven consumption

**Fig. 4:**
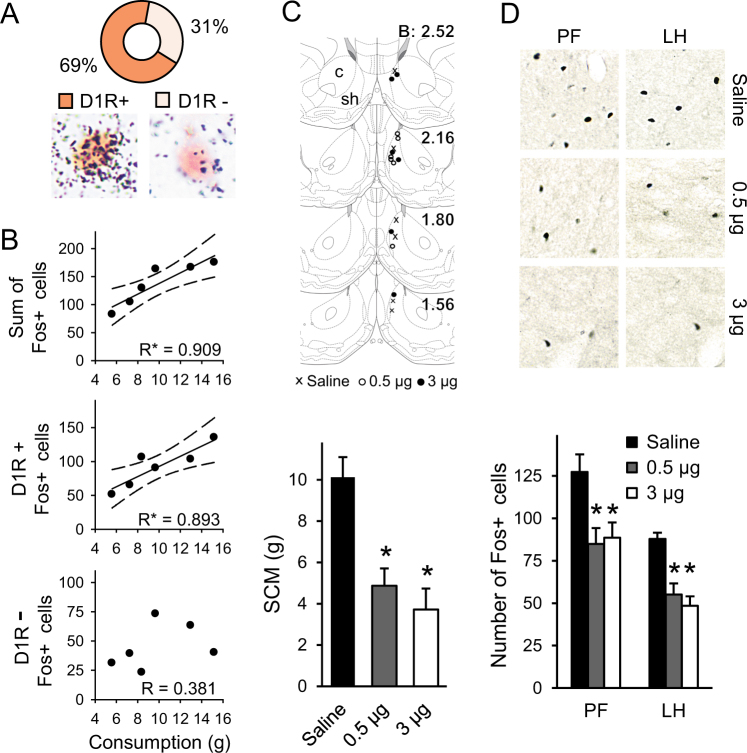
Evaluation of role of medial shell D1R signaling in reward–related food intake. **a** Sweet milk intake elicited neuronal activation mostly in D1R+ and less in D1R− cells in the medial shell. The representative pictures show Fos-immunoreactive cells (brown nuclei) with positive (left, dense silver grain accumulation) and negative (right, few scattered grains at background level) D1R mRNA labeling. **b** Fos activation in the accumbens nucleus in relation to the amount of consumed sweet milk. As before (Fig. 2b), a strong correlation was established between the consumed quantity and the number of Fos+ neurons. Separate analysis of D1R-positive and D1R-negative Fos cells verified that only D1R-bearing neurons code reward-related information. Solid and dashed lines show linear regressions and 95% confidence intervals, respectively, see correlation coefficients on the graphs, *p < 0.05, *n* = 6. **c** Influence of local stimulation of medial shell neurons with D1 agonist SKF-82958 on SCM intake and neuronal activation in the LHA. Top: Illustration of microinjection sites for different doses of SKF-82958. The diagrams were adopted from the Atlas of Paxinos and Watson [24] and illustrate frontal section levels rostral to Bregma in mm as indicated. Bottom: SCM consumption was attenuated markedly by the treatment independently from doses applied. **d** The number of Fos+ cells in the lateral hypothalamic area after SCM intake. The different doses reduced cell activation in a similar manner in both investigated areas. Top: Fos+ cell nuclei appear as black dots on the representative pictures. Bottom: Results of the quantitative analyses, *n* = 6,*p < 0.05 vs. saline. Data are presented as means ± SEM. c core, sh shell, LH lateral hypothalamic, PF perifornical regions of the lateral hypothalamic area

As the relationship between the SCM-evoked Fos-activation and D1R expression of mSh neurons remained an open question, in a repeated experiment we challenged normally nourished rats with SCM, and assessed colocalization of D1R mRNA and Fos-immunoreactivity in the mSh. More than two-thirds of the Fos+ neurons (69 ± 4 %) expressed D1R mRNA (Fig. 4a). Again, a strong linear correlation was apparent between the quantity of SCM consumed and the number of Fos+ cells (*R* = 0.909; *p* < 0.05) (Fig. 4b). However, when the D1R-positive and negative Fos populations were analyzed separately, it became clear that only the D1R-expressing neurons reflect reward-related food intake proportionally to consumed SCM quantity (*R*_D1Rpos_ = 0.893; *p* < 0.05 and *R*_D1Rneg_ = 0.381; *p* = 0.456) (Fig. 4b).

To address the function of D1Rs in feeding reward, a selective D1R agonist, SKF-82958, was infused into the right mSh of normally nourished rats (Fig. 4c, top), just before palatable food intake. The doses applied (0.5 and 3 µg) attenuated greatly the amount of consumed SCM (*p* < 0.01) (Fig. 4c, bottom) and duration of the meal (*p* < 0.05) (Table 2), without significant differences between the doses. In agreement with the reduced consumption, stimulation of mSh D1Rs also led to a decreased number of SCM-evoked Fos+ neurons in the LHA. The two doses acted similarly, and the response was obvious in both measured regions (*p* < 0.05 for PF and, *p* < 0.001 for LH) (Fig. 4d). However, the treatments did not influence liking-associated reactions during consumption, such as initial and mean licking rates, mean cluster size or duration (Table 2).

**Table 2:** Effect of D1R agonist (SKF-82958) infusion into the right medial shell on the microstructure of consummatory behavior

	Meal duration (s)	Initial licking rate (1/s)	Mean licking rate (1/s)	Mean cluster size (licks)	Mean cluster duration (s)
Saline	402 ± 29	6.6 ± 0.1	6.5 ± 0.1	68.3 ± 5.9	10.5 ± 0.8
0.5 µg SKF-82958	232* ± 42	7.0 ± 0.3	6.6 ± 0.2	59.9 ± 9.5	9.2 ± 1.4
3 µg SKF-82958	181* ± 50	7.3 ± 0.1	6.9 ± 0.1	52.4 ± 7.9	7.5 ± 1.0

Data are presented as means  ±  SEM, *n* =  6, *p  <  0.05 vs. saline

## Discussion

The role of the Nac-LHA circuit has recently been emphasized in cessation of the eating behavior, which is crucial to prevent overeating [13, 20, 39]. Perturbation of function may cause abnormal eating behavior by an unknown mechanism.

We investigated this circuit in normal, as well as in PR rats, as it was shown earlier that prenatal protein or general food restriction leads to hyperphagia and an altered response to food reward [4–7]. The PR feeding model we used here is a good representation of the human situation for generating a late onset obese phenotype with metabolic syndrome [40], although development of the condition greatly depends on the postnatal nutritional supply [41, 42]. Generally, late catch-up growth and high caloric food exacerbate the manifestation of obesity [4, 41], while the lack of these factors delays it [42, 43]. Our subjects were young adults fed with standard rat chow from weaning. Catch-up growth started from the 2nd week, as the pups with intrauterine retardation become stronger, but definitive hyperphagia was observed only from the 11th week. We chose 14-week-old rats for the experiments because by that time a slight and constant hyperphagia has developed and the bodyweight of the animals was in the normal range. The intrauterine protein restriction during gestation only has been reported to produce rats with a normal lipid profile, glucose & insulin concentrations compared to those of controls up to the age of 9 months [41, 43]. The weights of fat depots are also comparable to controls in these young adults [44, 45]. This indicates that metabolic regulation has the capacity to keep the parameters in balance at young ages, and thus the alterations we found precede the metabolic changes, and do not happen along with them.

When we evaluated the attitude for food reward, PR rats licked significantly more SCM solution, even during the training period. PR rats in an operant conditioning task produced higher response rates compared to controls [6], suggesting an increased motivation. Since ‘liking’ and motivation together determine consumption, we assessed “liking” reactions [25, 46]. We failed to detect elevated “liking” reactions in PR rats. Thus, the increased motivational aspect of reward is probably the only reason behind the higher consumption.

Sweet taste represents a high reward valence and triggers a robust neuronal activation in the Nac [47]. Based on neuronal activation-induced Fos expression, we identified a reward-reflecting D1R-bearing neuronal population in the mSh both in control and PR rats. Quite remarkably, activation of these neurons is proportional to the quantity of SCM consumed. The amount of activated cells may depend on the amount of dopamine released, which may happen, as for example dopamine release occurs proportionally to the concentration of sucrose provided to rats [48, 49]. Higher SCM consumption, however, did not evoke a higher number of Fos+ cells in PRs, as it would be expected if intensified responsiveness for feeding reward was assumed. Instead, mSh reward-reflecting neurons displayed reduced sensitivity for reward. Additionally, D1R deficiency appeared in the mSh of PRs, suggesting that a shortfall of D1R signaling may be responsible for these findings. Since there was no sign of elevated dopamine synthesis in the VTA, D1R downregulation did not occur secondarily in response to augmented dopamine release. According to the revised reward deficiency hypothesis for overeating, dopamine deficiency is a primary factor in driving reward-seeking behavior [50], and both D1Rs and D2Rs in the Nac endorse addiction-related behaviors [51]. D2R expression was unaffected in PRs, as we expected, considering that PR rats were lean, and that the generation of striatal D2R deficiency is a leptin-dependent process [8, 9, 12]. On the other hand, D1R expression is independent from leptin [12] and negatively regulated by the satiety signal, ghrelin, a hormone potent in food reward reinforcement [52]. Elevated plasma ghrelin levels have been found perinatally in different intrauterine undernourished rat models [53, 54] and in newborn infants with intrauterine growth retardation [55].

D1R+ neurons in the mSh control feeding behavior. D1R stimulation inhibits GABAergic neurons in the rostral LHA through a direct projection, resulting in the cessation of feeding [20]. By using local D1R agonist injection into control rats, we showed that D1R stimulation enhances the attainment of reward satisfaction, and also reduces hedonic food consumption. There is an orexigenic GABAergic cell population in the rostral LHA that is immunonegative for both MCH and orexin, the stimulation of which enhances both appetitive and consummatory behaviors [21, 22]. These are probably the targets of the mSh D1R+ cells, as in our experiments D1R stimulation suppressed the SCM-evoked activation of the rostral LHA non-MCH, non-orexin neurons in both measured regions. Conversely, in D1R-deficient PR animals, the control of LHA orexigenic neurons was impaired; they had an elevated number of Fos+ cells in the LHA, and consumed more SCM than their normally nourished peers. We observed a difference in reaction between the measured LHA regions, suggesting a difference in sensitivity between the lateral and perifornical populations, which confirms that LHA reward-responsive cells are heterogenous [22]. Interestingly, SCM drinking induced Fos in the orexin cells medial to the fornix. These cells probably reacted to dopamine released from VTA during SCM drinking. Medial orexin cells lack D1-2Rs, and are transsynaptically regulated by dopamine [56]. Since they are not innervated by the Nac, they were not in focus regarding the present study. However, the role of these cells was revealed in dopamine agonist-elicited arousal [56].

In summary, we propose that dopamine released upon SCM drinking proportionally builds up a stop signal in D1R-bearing mSh neurons that finally culminates as reward is satieted, and leads to the termination of food intake through the inhibition of LHA cells. A D1R deficiency induces an increased threshold of reward-reflecting neurons, shifting the level of termination. Future directions would include clarifying how exactly phenotypical changes occur, and particularly whether accumbal D1R deficiency could be connected to the development of striatal D2R downregulation later on. A more challenging question is how prenatal programming could be reversed in order to maintain a healthy reward system.

## Electronic supplementary material

Supplementary table 1

Supplementary table 2

Supplementary table 3
